# Optimisation and *In Vivo* Evaluation of Pectin Based Drug Delivery System Containing Curcumin for Colon

**DOI:** 10.1155/2014/924278

**Published:** 2014-07-02

**Authors:** Kishor Butte, Munira Momin, Hemant Deshmukh

**Affiliations:** ^1^Department of Pharmaceutics, Oriental College of Pharmacy, Sector 2, Sanpada, Navi Mumbai 400 705, India; ^2^Department of Radiology, Seth GS Medical College and KEM Hospital, Mumbai 400 012, India

## Abstract

The higher incidences of side effects of existing drugs have shifted researchers and clinicians to explore the dietary phytoconstituents for its therapeutic potentials. The present study is based on compression coated curcumin tablet for the colon. Curcumin has anti-inflammatory and antioxidant properties. Curcumin presents a bioavailability problem due to poor solubility. An inclusion complex was formed with hydroxypropyl-*β*-cyclodextrin to enhance the solubility. In this study, the core tablet of curcumin inclusion complex was compressed between the layers of polymer blend of pectin and Eudragit S100. The 3^2^ full factorial design was utilised for optimization of the formulation. The polymer ratio (*X*1) and coat thickness (*X*2) presented significant effects on the selected responses, i.e., percent drug release after 4 hours (*Y*240) and difference in percent drug release between 4th and 6th hour (*Y*_diff_) in presence of pectinase enzyme. The results revealed that higher coat weight (600 mg) and higher level of pectin ratio (70% w/w) protected the curcumin tablet till ascending colon. The* in vivo *studies by roentgenography method using human volunteers supported these observations. Hence, it can be concluded that the combination of pectin and Eudrgit S100 makes the system biodegradable and pH dependent for targeting the drug to the colon.

## 1. Introduction

Colon targeting is one of the approaches which can be used for local and systemic delivery of drug. Colonic drug delivery system (CDDS) has gained increased importance not only for the delivery of the drugs for the management of diseases associated with the colon like Crohn's disease but also for the systemic delivery of proteins and therapeutic peptides. The large intestine is difficult to reach by peroral delivery, but it is still deemed to be the ideal site for the delivery of drugs to cure the local diseases of the colon [1]. The most critical challenge in such drug delivery approach is to protect the formulation during its passage through the stomach and upper intestine. Several approaches have been studied to achieve colonic targeting including pH dependent system and biodegraded system [2–5]. The pH approach suffers drawback of premature release of drug due to unpredicted change in pH of the GI tract. The bacterial degradation of natural polymers in the colon is a well-established approach [6–9]. A large number of polysaccharides are degraded by colonic bacteria and may form the basis for a suitable carrier. Pectin is one such heterogeneous polysaccharide present in the cell wall of the plants. It consists mainly of D-galacturonic acid and its methyl ester linked via *α*-D(1→4) glycosidic bonds. Various reports suggest that pectin and its salt may be of value in designing drug delivery formulation to the colon [10–13]. It is nontoxic and almost totally degraded by colonic bacteria and is not digested by gastric or intestinal enzymes. A constraint of the polymer, pectin is its high solubility, but this can be easily overcome either through choice of high methoxylated pectin or presence of additives. The pH erosion controlled system with polymers like Eudragit and its different grades can be a promising approach for releasing drug only to the colon.

The present investigation is based on development of colon targeted drug delivery system containing curcumin as an active phytoconstituent. Curcumin has antioxidant, anti-inflammatory, anticarcinogenic, antimicrobial, antispasmodic, antiviral, and hepatoprotective activities. Curcumin is derived from the rhizomes of turmeric (*Curcuma longa Linn*.), which is a member of the ginger family (*Zingiberaceae*). Curcumin also inhibits human colon cancer cell growth which is mediated via signaling cascades including the modulation of the NF-*κ*B signaling pathway. Curcumin increases the levels of other endogenous antioxidants through the Nrf2 pathway to strengthen body's defence against reactive oxygen species (ROS) [14]. Due to its anti-inflammatory and antioxidant action of curcumin, it is an ideal candidate for development of colonic drug delivery system. Despite its clinical effectiveness, the poor oral bioavailability restricts its usage in oral dosage form. The present study also includes formation of an inclusion complex of curcumin with well-studied cyclodextrins.

Compression coated tablet is a promising approach due to its thicker coatings as compared to a traditional film coating processes which is also time-consuming and has possibilities of giving premature drug release. The added advantage of thick coat of compression coat can further protect the core till it reaches the colon.

A 3^2^ full factorial design was employed in this study to systematically design and develop colon specific curcumin tablets using pectin and Eudragit S100 for compression coating [15]. The roentgenography method is one of the methods to track the tablet in the body by taking X-rays at different time intervals. In this method, the drug of the core can be replaced with X-ray opaque material like barium sulphate. The movement of the tablet as well as its capacity to protect the core of the tablet was studied in healthy human volunteers.

## 2. Materials

Curcumin was purchased from HiMedia Laboratories Ltd., India. Pectin was purchased from SD Fine Chemicals, Mumbai, India, hydroxypropyl-*β*-cyclodextrin was gifted by Roquette Pharma, Mumbai, India, and Eudragit S100 was gifted by Evonik Degussa Pvt. Ltd., India. All other reagents were of analytical grade and used as received. Double distilled water was prepared freshly.

## 3. Method

### 3.1. Preparation of Curcumin Inclusion Complex Core Tablet

An inclusion complex of curcumin was prepared using hydroxypropyl-*β*-cyclodextrin (HP*Β*CD) as reported earlier by Singh et al. [16]. In brief, a required quantity (100 mg) of curcumin and HPBCD was weighed accurately in 1 : 0.5, 1 : 1, and 1 : 2 molar ratios. The complex was prepared by kneading method. The effectiveness of molar ratio of curcumin-HP*Β*CD inclusion complex was studied by the saturation solubility study.

Core tablet composed of 126 mg of curcumin-HP*Β*CD complex equivalent to 100 mg of curcumin was compressed using microcrystalline cellulose (5% w/w), cross-povidone (2% w/w), and citric acid (2% w/w). All the excipients were sifted through 40# sieve and blended together for 20 minutes and lubricated for 5 minutes using magnesium stearate (1% w/w) and talc as a glidant (2% w/w). Core tablets were compressed by using 8 mm bevelled punch by the direct compression method and were evaluated for parameters like appearance, dimensions, hardness, disintegration time, assay, uniformity of weight, and content uniformity.

### 3.2. Gelling and Swelling Study of Polymer Blend

Pectin and its physical blend with Eudragit S100 in varying proportions (100 : 0, 70 : 30, 50 : 50, and 30 : 70) were subjected to gelling and swelling study in 0.1 N HCl, PBS 6.8, and PBS 7.4. Gelling and swelling were observed after standing for 24 h.

### 3.3. Optimization of Compression Coated Tablet Using 3^2^ Full Factorial Design

A 3^2^ full factorial design was adopted to optimise the selected formulations variables. Based on swelling and gelling study of polymers, different ratios of pectin and Eudragit S100 (*X*1) were selected, that is, 30 : 70, 50 : 50, and 70 : 30. The second independent variable selected was the effect of the coat thickness on drug release profile (*X*2), that is, 400 mg, 500 mg, and 600 mg. Based on 3^2^ factorial designs total of nine batches was proposed. The formulations variables and their levels are shown in Table 1. The dependent variables chosen were drug release at 4th hour (*Y*240), difference in percent drug release between 4th and 6th hour (*Y*_diff_), and total drug release after 6 hours (*Y*_Total_) in presence of pectinase enzyme.

### 3.4. Compression Coating of Curcumin Core Tablets

Compression coating for all nine formulations was carried out by the direct compression method using the previously compressed and evaluated core tablet. Briefly, less than a half of the die cavity was first filled with the blend of coating polymers. The previously compressed curcumin core tablet was then placed in the center of the die cavity carefully. The die was then completely filled with remaining coating polymer blend. Compression was carried out using 12.7 mm bevelled punch set on rotary tablet press (Cadmach Machinery, India). All the formulations were then subjected to pharmacotechnical evaluation and* in vitro* dissolution studies.

### 3.5. *In Vitro* Dissolution Testing of Compression Coated Curcumin Tablets

*In vitro* dissolution study was carried out using pH 1.2 buffer (2 hrs), phosphate buffer saline 6.8 (2 hrs), and pH 7.4 phosphate buffer saline (PBS) containing 1% SLS as dissolution media. The present formulation is pH dependent and biodegradable system. The curcumin compression coated tablets contain pectin which undergoes enzymatic degradation in the colon. In order to mimic colonic conditions and ensure maximum drug release, PBS 7.4 containing 3% pectinase enzyme was used after 4th hr. The addition of pectinase enzyme mimics the colonic conditions. Dissolution was carried out using USP Apparatus II (paddle) at 50 rpm and absorbance was measured by UV spectrophotometer at 425 nm.

### 3.6. *In Vivo* Roentgenography Studies

Roentgenography study was carried out to access the* in vivo* performance of the formulation. Similar reports have been made by one of the authors for studying the performance of colon targeted drug delivery system using pH sensitive and biodegradable system [17]. The main purpose of this study is to track the movement, integrity, and disintegration of the tablet in the GIT. The studies were carried out using barium sulfate as X-ray opaque material by replacing it with the core of the curcumin inclusion complex. Each core tablet for* in vivo* study consists of 126 mg of barium sulfate equivalent to drug complex and concentration of all other excipients was kept the same. Barium sulphate core tablet was compressed using same hardness, friability, and thickness as that of curcumin tablet. This core tablet was then compression coated using the optimized blend of pectin and Eudragit S100.

To perform the Roentgenography studies, permission from human ethical committee of KEM hospital, Mumbai, India, was taken and the study was performed at the Radiology Department of KEM Hospital, Mumbai, India. A written consent was taken from the volunteers before performing the study. Healthy 6 male volunteers between the age group of 18 and 29 years participated in the study. Volunteers were nonsmokers, nonalcoholics, and not on any drugs. On previous night bowel cleansing preparation was given to the volunteers. After overnight fasting, each volunteer orally ingested compression coated barium sulfate tablet with 500 mL of water. X-ray of GIT was taken at intervals of 0 hr, 2 hr, 4 hr, 6 hr, 8 hr, and 24 hr to follow the movement, location, and integrity of the tablet in GIT.

### 3.7. Stability Studies of Compression Coated Tablets

As per ICH guidelines, tablets of the optimized formulation were tested for stability under three conditions for a period of three months. The tablets were packed in suitable container and stored in stability chambers maintained at 25°C/60% RH, 30°C/65% RH, and 40°C/75% RH and were evaluated for their physical characteristics,* in vitro* release, and content of curcumin at the end of 1 month, 2 months, and 3 months of storage period.

## 4. Results and Discussions

An inclusion complex of curcumin with HPBCD was prepared. Based on the results of solubility improvement, 1 : 1 ratio of curcumin to HPBCD was further selected. Core tablets of curcumin inclusion complex were compressed using direct compression method. All the tablets showed less than 15 seconds disintegration time. The curcumin core tablet batch passed all the quality control parameters. Core tablets had average thickness of 8.01 ± 0.02 mm and average weight of 140.45 ± 1.10 mg.

Gelling and swelling studies revealed that pectin alone does not form good gel and hence is not a candidate to be used alone for compression coating. The pH dependent polymer, Eudragit S100, was added in varying proportion. The polymer blend showed effect on gelling and swelling. During this study, it was proposed that 30% to 70% ratio of pectin to Eudragit at various level can be further explored for optimization of the final formulation. It was concluded that the ratio of polymers will play an important role to get a desired drug release profile.

Compression coating of the tablet was done by using different polymer ratios (*X*1) and coat weight (*X*2) as per the 3^2^ full factorial design. All the nine batches were found stable up to 3 months when subjected to different environment as per the ICH guidelines.

**Figure 1:**
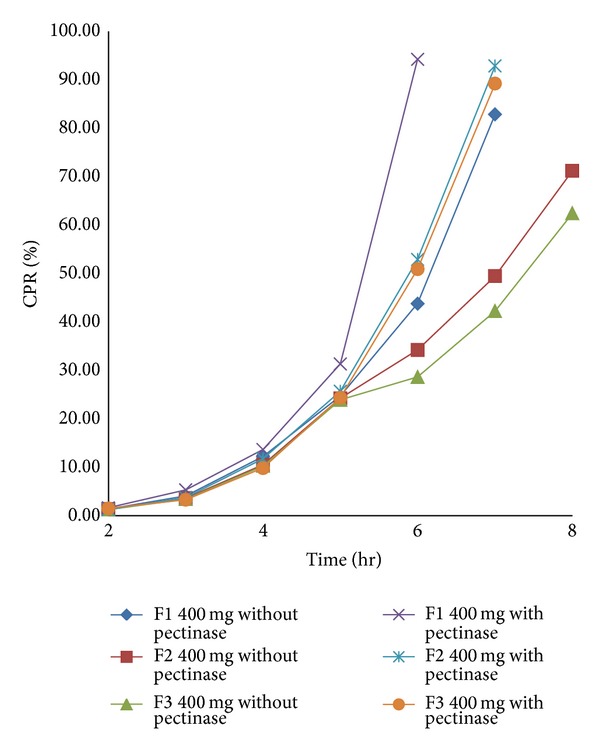
*In vitro* dissolution profile of batch F1 to F3 with and without pectinase enzyme.

**Figure 2:**
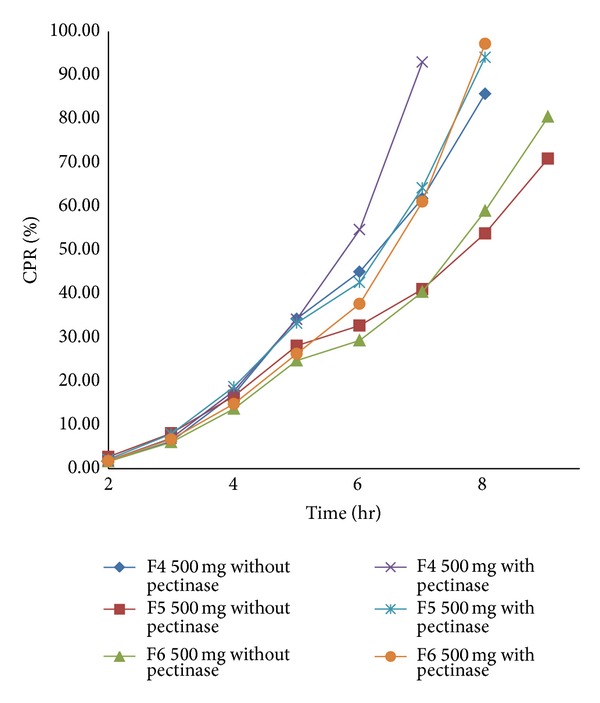
*In vitro* dissolution profile of batch F4 to F6 with and without pectinase enzyme.

**Figure 3:**
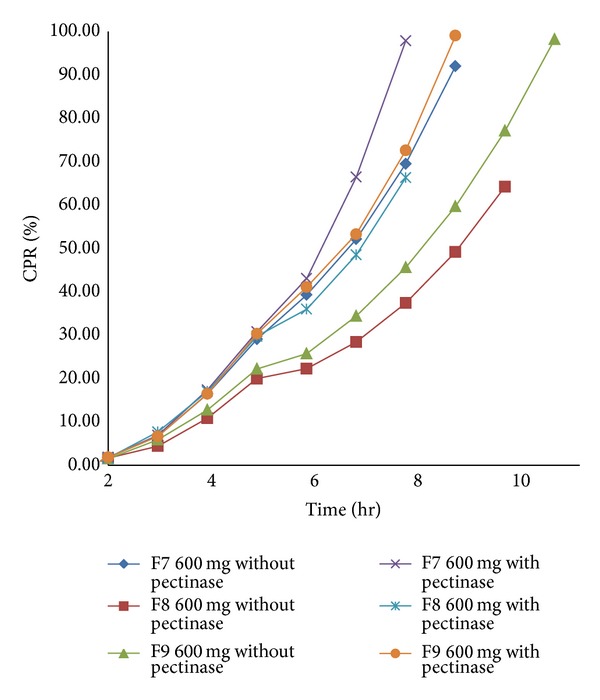
*In vitro* dissolution profile of batch F7 to F9 with and without pectinase enzyme.

For the present investigation, it is desirable to design the formulation such that it prevents drug release in upper GIT and release curcumin content only in the colon by undergoing bacterial degradation of pectin of the compression coat. To determine the bacterial degradation of pectin coat, dissolution was carried out with and without 3% w/v pectinase enzyme. The pectinase in mentioned quantity was added at the 4th hour to simulate the colon arrival time under normal conditions.* Y*240 and *Y*_diff_ of all batches are shown in Table 1. Comparative dissolution profile of batches F1–F9 shown in Figures 1 to 3 indicates that as the amount of pectin increases, drug release in the initial hours can be retarded. *Y*_diff_ values for the nine batches showed a wide variation; that is, the response ranges from a minimum of 22.89% to 80.55%. At the lower level of pectin, degradation of the tablet was fast and hence premature drug release was observed. Higher pectin levels (batches F7 to F9) showed slower drug release.* Y*240 values were found to be 17.40%, 17.05%, and 16.50% and *Y*_diff_ values were found to be 25.68%, 18.97%, and 24.59%, respectively. This indicates that the drug release is very slow due to stiff gel formation, which could not be degraded by bacterial enzymes. The formulation was intended to release most of the curcumin between 7th hour and 9th hour. Batch F9 was selected as optimized formulation as the *Y*_diff_ is minimum, which was desirable. From the overall dissolution data it can be concluded that pectin plays an important role in the optimization of the formulation. The* in vitro* dissolution data in the absence and presence of pectinase enzyme are shown in Figures 1, 2, and 3.

A full 3^2^ factorial design was applied to optimize the compression coated tablets of curcumin using pectin and Eudragit S100. The amount of pectin in coat (*X*1) and coat weight (*X*2) was used as independent variables. Dependent variables chosen were* Y*240 and *Y*_diff_. The following polynomial equation was derived for* Y*240 response:

(1) Y240=+15.29−3.53X1 +1.84X2+1.02X12+0.56X22(P<0.005,R2=0.963).

The above equation reveals that the negative sign of factor* X*1 can be explained as increase in the pectin concentration which leads to reduction in the* Y*240. This is because when pectin comes in contact with colonic fluid, it starts degrading in the presence of colonic microbial flora. This will lead to exposing the core tablet and releasing the drug only after complete biodegradation. The higher concentration of the Eudragit in the coat will lead to a higher release of the drug at upper intestinal pH.

**Figure 4:**
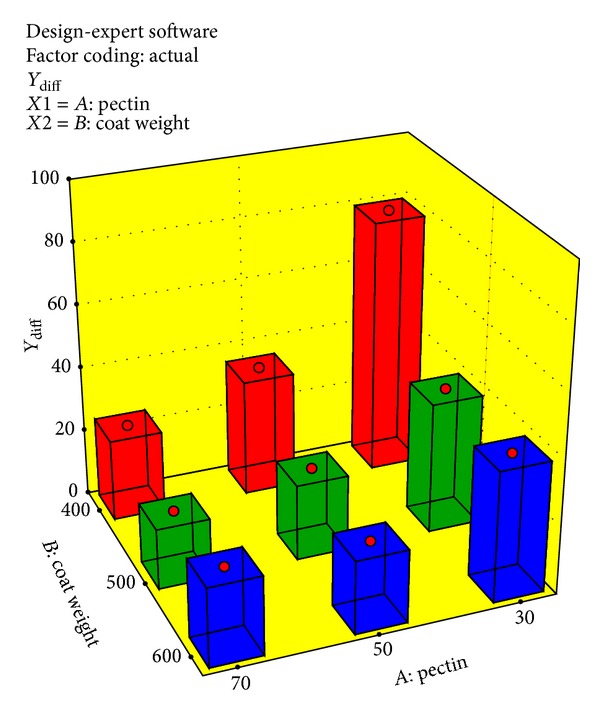
Effect of pectin and coat weight on *Y*_diff_.

As per ANOVA results, *Y*_diff_ response is significant at selected independent variable while *Y*_Total_ did not show significant results. This is very much true in the philosophy of the formulation development for colonic drug delivery using natural polymer and pH dependent polymer. After 4th hour, the tablet enters into the colonic region where microbial flora will attack the natural polymer and also the higher pH will lead to the dissolution of pH dependent polymer. Hence, after 6th hour, mainly the coat weight of the compression coated tablet plays a vital role.  Figure 4 depicts a clear picture of effect of formulation variables on the response selected.

Hence, the batch F9 was selected an optimized batch for further* in vivo* performance test by roentgenography considering that higher coat weight and higher pectin ratio will make the formulation biodegradable system.

**Figure 5:**
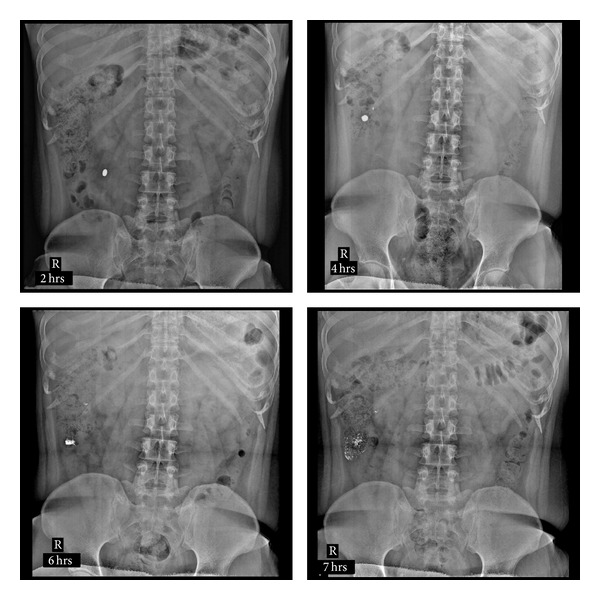
X-ray slides showing compressed coated tablet at different time interval in healthy human volunteer.

The compression coated tablet of barium sulfate was ingested by the healthy volunteers and X-rays were taken at different time intervals, that is, 0 hr, 2 hrs, 4 hrs, 6 hrs, 7 hrs, and 24 hrs. All volunteers showed almost the same results. The X-ray images of volunteer 3 are shown in Figure 5. The colonic arrival time of the tablet was 3-4 hr. It was observed that the tablet was swollen but remained intact till 4th hour. Barium sulfate was not released either in the stomach or small intestine of the volunteers. At 6th hour, in the colon the tablet was seen little disintegrated. The 7th hour X-ray slide showed quick spillage of barium sulfate in ascending colon which proves that on entering the colon, the coating of the tablet began to degrade because of bacterial enzymatic action. The process of degradation of pectin appears to have initiated at around 6th hour after ingestion of tablet.

The above results indicate the efficiency of the formulation F9 to protect the core till it reaches the colon. The 24th hour X-ray slide showed few opaque particles. Hence, it was clear that formulation F9 will remain in colon up to 24 hrs. Curcumin having low solubility will remain in colon till 24 hrs which helps in systemic as well as localized action.

## 5. Conclusion

Curcumin is a potent drug molecule having antioxidant, anti-inflammatory, and anticarcinogenic activity. Curcumin is insoluble in water. In order to increase solubility, stability, and absorption, inclusion complex of curcumin with hydroxypropyl-*β*-cyclodextrin (HP*β*CD) was formed. Hydroxypropyl-*β*-cyclodextrin (HP*β*CD) enhances the solubility of curcumin. Pectin being biodegradable polymer gets degraded in colonic enzymes while Eudragit S100 gets solubilized at pH 7.0. Both these polymers have capacity to protect the core in the upper GIT and helps in achieving targeted release of curcumin in the colon, which was supported by* in vitro* dissolution study. Roentgenography study further proved the performance of optimized formulation to protect the core till it reaches the colon.

## Figures and Tables

**Table 1:** 3^2^ factorial batches with their variables.

Trials	Independent variables				Responses		
	Coded		Actual		*Y*240	*Y*_diff_	*Y*_Total_
	*X*1	*X*2	*X*1	*X*2			
					13.67	80.55	94.23
F1	−1	−1	30	400	11.75	41.17	52.92
F2	0	−1	50	400	9.84	41.07	50.92
F3	1	−1	70	400	17.85	36.84	54.68
F4	−1	0	30	500	18.75	23.84	42.60
F5	0	0	50	500	14.80	22.89	37.69
F6	1	0	70	500	17.40	25.68	43.08
F7	−1	1	30	600	17.05	18.97	36.02
F8	0	1	50	600	16.50	24.59	41.09
F9	1	1	70	600	13.67	80.55	94.23

*X*1: polymer ratio (pectin); *X*2: compression coat weight; *Y*240: percent drug release after 4 hours; *Y*_diff_: difference in percent drug release between 4th and 6th hour; *Y*_Total_: total percentage of drug release after 6 hours.
